# Prevalence of apical periodontitis in root-canal-filled teeth – a cross-sectional study

**DOI:** 10.2340/aos.v85.46663

**Published:** 2026-08-13

**Authors:** Nikki Savolainen, Thomas Kvist, Helena Fransson, EndoReCo, Fredrik Frisk

**Affiliations:** a School of Health and Welfare, Jönköping University, Jönköping, Sweden; b Department of Endodontology, Institute for Postgraduate Dental Education, Jönköping, Sweden; c Department of Endodontology, Institute of Odontology, The Sahlgrenska Academy, University of Gothenburg, Gothenburg, Sweden; d Department of Endodontics, Faculty of Odontology, Malmö University, Malmö, Sweden; e The researchers within the Endodontic Research Collaboration in Scandinavia contributed to this study

**Keywords:** Cross-sectional studies, periapical periodontitis* / epidemiology, prevalence, root canal obturation, root canal therapy

## Abstract

**Objective:**

The aim of this cross-sectional study was to assess the root canal filling quality and the frequency of apical periodontitis (AP) in root-canal-filled teeth in a randomly selected adult non-patient population in Sweden.

**Materials and Methods:**

In 2023, 295 root-canal-filled teeth were identified in 126 adults in a cross-sectional study in Jönköping, Sweden. Periapical radiographs were assessed by two calibrated observers. The periapical status was scored according to the periapical index (PAI) and then dichotomized as either healthy (PAI 1–2) or diseased (PAI 3–5). The root canal filling quality was deemed as adequate/inadequate. An adequate root canal filling was considered to end 0.5–2 mm from the radiographic apex and to have no radiolucent voids.

**Results:**

Adequate root canal fillings were seen in 89 (30.2%) of the teeth. AP was present in 94 (31.9%) of the teeth, and of these, 72 (76.6%) had an inadequate root canal filling. Of the 122 root-canal-filled molars, 101 (82.8%) had inadequate root canal filling and 57 (46.7%) had AP.

**Conclusion:**

A large proportion of root-canal-filled teeth have inadequate root canal filling quality and AP, especially in molars. Both contributing factors and strategies for improvement require further study.

## Introduction

Root canal treatment (RCT) aims to treat and prevent infection and inflammation in the dental pulp and periapical tissues [1]. The success of RCT is usually assessed as the absence of clinical and radiological signs of apical periodontitis (AP) at post-treatment controls [1–3]. Many factors affect the outcome; for example, the technical quality of the root canal filling is associated with periapical healing [2, 4–6]. Because of this, new methods and techniques are constantly being developed to achieve better treatment outcomes [7, 8]. However, in contemporary studies of general populations, the frequency of AP in root-canal-filled teeth remains high, approximately 30–45% [5, 9–11].

In Jönköping, Sweden, a cross-sectional study showed AP in 34.0% of the root-canal-filled teeth in 2013 [11]. This was an increase in the frequency of AP in root-canal-filled teeth in comparison to a previous cross-sectional study in Jönköping in 2003 [12]. The frequency of AP was highest among root-canal-filled molars, of which 46.0% had AP. Molars were also the largest tooth group in the sample, and a large proportion of molars among root-canal-filled teeth has been seen in other cross-sectional studies as well [10]. In addition to the increase in the frequency of AP, the frequency of technically inadequate root canal fillings increased between 2003 and 2013, with 71% of root-canal-filled teeth having technically inadequate root canal filling quality in 2013 [11]. High frequency of inadequate root canal filling quality has been seen in several cross-sectional studies [13–15].

Over time, changes in the population may affect overall oral health and treatment needs. In Sweden, the general oral health has improved during the last decades [16]. Consequently, the Swedish population retains more teeth, including more molars, in higher ages [16]. The aging population contributes to new challenges for the dental care system, since ageing is associated with increased risk for poor oral health [17]. There have also been changes in the general population in Sweden due to the increased immigration [18], which in turn, can affect the general oral health. Importantly, while the population structure has faced changes after 2013, there has also been limited access to dental services in certain geographic areas during the past decade [19], and this may have caused an accumulated treatment need. A decrease in the number of RCTs has been observed both in Sweden [16] and internationally [10], while the number of implant treatments has increased in Sweden [20]. This may represent a shift in treatment strategy among Swedish dentists concerning RCT.

Thus, given the changes described affecting the general population, it remains important to carefully monitor the prevalence of AP in root-canal-filled teeth. Since cross-sectional studies with the same sample criteria and study method have been conducted every 10 years since 1973 in Jönköping, there is a unique opportunity to study the development of the frequency of AP in root-canal-filled teeth and root canal filling quality in a non-patient-based population. This is especially relevant now, since the 2013 study was the first in the study series to deviate from the previously observed trend towards improved RCT outcomes [11, 12]. The aim of this cross-sectional study was to assess the root canal filling quality and the frequency of AP in root-canal-filled teeth in a randomly selected adult non-patient population in Jönköping, Sweden.

## Material and methods

The data collection was part of a larger cross-sectional study that was conducted in Jönköping between spring 2023 and fall 2024. An ethical approval application was sent to the *Swedish Ethical Review Authority* and was approved prior to the commencement of the study (Dnr 2022-04308-01). The STROBE checklist was used when writing the manuscript [21].

The study sample consisted of adult individuals of 20, 30, 40, 50, 60, 70, and 80 years old in 2023, living in four parishes in the city of Jönköping. No other inclusion or exclusion criteria were applied. As in previous cross-sectional studies in the Jönköping study series [11, 12], among individuals following these criteria, 910 were randomly selected to participate in the study, with 130 individuals in each age category. The invitations were sent by letter with information about the study. The individuals accepting the invitation were asked to give written consent at the beginning of the examination. The signed consent forms were securely stored in a locked cabinet.

### Data collection

The examinations took place at the Institute for Postgraduate Dental Education in Jönköping, Sweden, and consisted of a clinical and a radiographic examination as well as the use of a questionnaire about general and oral health. The radiographic examination contained intraoral radiographs of varying number: participants who were 60–80-years old underwent a full-mouth intraoral radiographic examination. Participants who were 30–50-years old had bitewing radiographs taken of the posterior teeth, as well as apical radiographs of the anterior teeth. Supplementary apical radiographs were taken on the posterior teeth in case of root canal fillings or visible pathology, for example a deep carious lesion. In the case of 20-year-old participants, bitewing radiographs were taken of the posterior teeth, as well as supplementary apical radiographs if a root canal filling was suspected to be found (e.g. in the case of a lingual composite filling on an anterior tooth) or if there was visible pathology, for example a deep carious lesion or a deep periodontal pocket. The intraoral radiographs were taken using KaVo Focus X-ray unit (Gendex GXIO-770™; KaVo Dental, Biberach, Germany) and Schick 33 intraoral sensors (Dentsply Sirona; Bensheim, Germany) and analyzed using a HP E23 G4 FHD monitor (HP Inc., Palo Alto, CA, USA) with a resolution of 1920 x 1080 in the dental radiograph program Onepix, version 2.5.6 (Unident AB, Falkenberg, Sweden).

### Data registration

In the present study, only radiographic data from examinations that included root-canal-filled teeth were used. The statistical unit was the root-canal-filled tooth. All the radiographs were controlled for root-canal-filled teeth, and when present, the periapical status and root canal filling quality were assessed. The registrations were done in IBM SPSS Statistics for Windows, Version 31.0 (IBM Corp., Armonk, NY, USA) which was also used for statistical analyses. The assessments were performed by two calibrated observers (NS and FF) between February and May 2025.

The periapical status of the root-canal-filled teeth was assessed using the periapical index (PAI), where each tooth was scored on a scale of 1–5 [22]. In the case of multi-rooted teeth, the root with the highest PAI score was chosen to determine the score for the whole tooth. In addition to giving each tooth a PAI score of 1–5, the periapical status scores were also dichotomized into healthy (PAI 1–2) and diseased (PAI 3–5). Before analyzing the periapical status of the radiographs of the sample, the observers used 100 reference images to calibrate and make intra-observer reliability tests. Inter-observer comparisons were made based on cases from half of the sample. The observers compared their PAI score assessments based on these root-canal-filled teeth and discussed the ones where the assessments differed until agreement.

The root canal filling quality was assessed as either adequate or inadequate, and it was based on two factors: (1) Adequate length for the root canal filling meant that the root canal filling material in the intraoral radiograph ended within 0.5–2 mm of the tooth’s radiographic apex. In the case of inadequate length, the length was registered as either too long or too short. (2) An adequate seal was defined as a homogenous root canal filling without any voids between the root canal walls and the root canal filling material. Both length and seal needed to be adequate for the whole root canal filling to be assessed as adequate. As in the assessment of periapical status in multi-rooted teeth, the root with the highest PAI score was used to evaluate root canal filling quality for the tooth. If all roots had the same PAI score, the root with the lowest-quality root canal filling determined the overall root canal filling quality for the tooth. Before assessing the root canal filling quality of the sample, calibration and reliability tests were made on 30 randomly selected root canal fillings from the sample. Each observer (NS and FF) assessed root canal filling quality in 30 root-canal-filled teeth from all tooth groups individually. All 30 teeth were then jointly assessed, and in case of disagreement, the case was discussed until consensus was reached.

In addition to periapical status and root canal filling quality, information on the age, sex, number of missing teeth, number of root-canal-filled teeth, was registered for each participant. For participants who did not have any root-canal-filled teeth, age, sex, and number of missing teeth were registered.

### Statistical tests

For the reliability tests before registrations, reliability analyses were conducted and Cohen’s Kappa was calculated. After the registrations, the statistical analyses were conducted in IBM SPSS Statistics. To investigate if there was a statistically significant association between different tooth-specific factors and AP, a multiple General Estimation Equation (GEE) analysis was conducted with the dependent variable being AP. A multiple GEE was used to account for clustering of root-canal-filled teeth within individuals, as observations from the same individual are not independent. The root-canal-filled tooth was kept as the statistical unit throughout the analysis. The level of significance was set at 0.05.

## Results

**Figure 1:**
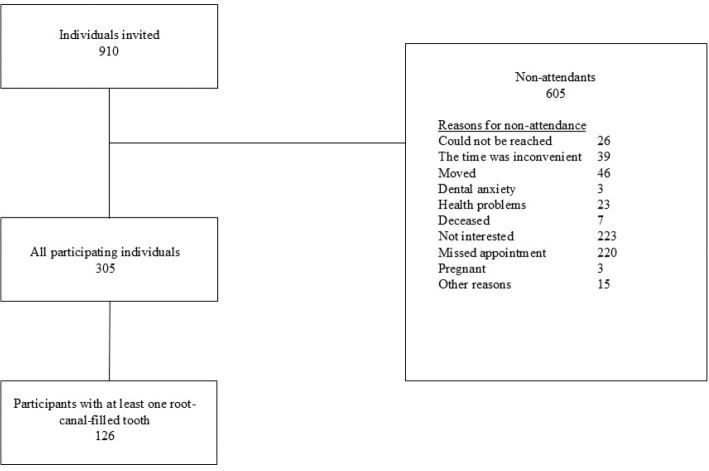
Flowchart showing the number of individuals at different stages of the study process. A total of 605 individuals did not attend, and the reasons for non-attendance were categorized into 10 groups.

In total, 910 individuals, 130 in each age group, were invited to participate in the study, of which 305 individuals (33.5% of the people invited) participated (mean age x̄ = 53.9; standard deviation [SD] = 18.3 years, mean number of teeth x̄ = 25.6; SD = 3.7). Of the individuals who did not attend, 49.3% were men and 50.7% were women (mean age x̄ = 48.1; SD = 20.5 years) (Table 1). There is no information on the dental status of these non-attendants. The reasons for non-attendance were recorded (see flow chart in Figure 1), of which missing the appointment for the examination as well as not being interested were the most frequent reasons. Of all the 305 participants, 126 had at least one root-canal-filled tooth (1–9 root-canal-filled teeth per individual), leading to a total of 295 root-canal-filled teeth that were examined radiographically and analyzed in this study. The average age among the participants with at least one root-canal-filled tooth was x̄ = 63.9; SD = 14.4 years. For the distribution of root-canal-filled teeth, adequate root canal filling quality, and AP in different age groups, see Table 2.

**Table 1:** Information of the participants and non-attendants regarding sex and age.

Variable	Individuals invited to participate, *n* (%)	Non-attendants, *n* (%)	All participating individuals, *n* (%)	Participants with at least one root-canal-filled tooth, *n* (%)
Total	910 (100.0)	605 (100.0)	305 (100.0)	126 (100.0)
Female	451 (49.6)	307 (50.7)	144 (47.2)	58 (46.0)
Male	459 (50.4)	298 (49.3)	161 (52.8)	68 (54.0)
Age group				
20	130 (14.3)	103 (17.0)	27 (8.9)	1 (0.8)
Female	72 (55.4*)	56 (54.4)	16 (59.3)	1 (100.0)
Male	58 (44.6*)	47 (45.6)	11 (40.7)	0 (0.0)
30	130 (14.3)	104 (17.2)	26 (8.5)	2 (1.6)
Female	56 (43.1)	46 (44.2)	10 (38.5)	0 (0.0)
Male	74 (56.9)	58 (55.8)	16 (61.5)	2 (100.0)
40	130 (14.3)	80 (13.2)	50 (16.4)	16 (12.7)
Female	57 (43.8)	36 (45.0)	21 (42.0)	9 (56.3)
Male	73 (56.2)	44 (55.0)	29 (58.0)	7 (43.8)
50	130 (14.3)	85 (14.0)	45 (14.8)	14 (11.1)
Female	60 (46.2)	34 (40.0)	26 (57.8)	8 (57.1)
Male	70 (53.8)	51 (60.0)	19 (42.2)	6 (42.9)
60	130 (14.3)	76 (12.6)	54 (17.7)	19 (15.1)
Female	61 (46.9)	37 (48.7)	24 (44.4)	10 (52.6)
Male	69 (53.1)	39 (51.3)	30 (55.6)	9 (47.4)
70	130 (14.3)	68 (11.2)	62 (20.3)	43 (34.1)
Female	69 (53.1)	40 (58.8)	29 (46.8)	16 (37.2)
Male	61 (46.9)	28 (41.2)	33 (53.2)	27 (62.8)
80	130 (14.3)	89 (14.7)	41 (13.4)	31 (24.6)
Female	76 (58.5)	58 (65.2)	18 (43.9)	14 (45.2)
Male	54 (41.5)	31 (34.8)	23 (56.1)	17 (54.8)

* % of people within this age category.

**Table 2:** Distribution of root-canal-filled teeth, adequate root canal filling quality and apical periodontitis (AP) based on sex and age group.

Variable	Root-canal-filled teeth, *n* (%)	Root-canal-filled teeth with adequate root canal filling quality, *n* (%)	Root-canal-filled teeth with AP, *n* (%)
Total	295 (100.0)	89 (100.0)	94 (100.0)
Female	134 (45.4)	41 (46.1)	34 (36.2)
Male	161 (54.6)	48 (53.9)	60 (63.8)
Age group			
20	1 (0.3)	0 (0.0)	0 (0.0)
Female	1 (100.0*)	0 (0.0)	0 (0.0)
Male	0 (0.0*)	0 (0.0)	0 (0.0)
30	2 (0.7)	0 (0.0)	1 (1.1)
Female	0 (0.0)	0 (0.0)	0 (0.0)
Male	2 (100.0)	0 (0.0)	1 (100.0)
40	26 (8.8)	7 (7.9)	10 (10.6)
Female	13 (50.0)	5 (71.4)	5 (50.0)
Male	13 (50.0)	2 (28.6)	5 (50.0)
50	38 (12.9)	12 (13.5)	14 (14.9)
Female	26 (68.4)	9 (75.0)	8 (57.1)
Male	12 (31.6)	3 (25.0)	6 (42.9)
60	29 (9.8)	7 (7.9)	6 (6.4)
Female	13 (44.8)	3 (42.9)	3 (50.0)
Male	16 (55.2)	4 (57.1)	3 (50.0)
70	100 (33.9)	33 (37.1)	37 (39.4)
Female	29 (29.0)	7 (21.2)	5 (13.5)
Male	71 (71.0)	26 (78.8)	32 (86.5)
80	99 (33.6)	30 (33.7)	26 (27.7)
Female	52 (52.5)	17 (56.7)	13 (50.0)
Male	47 (47.5)	13 (43.3)	13 (50.0)

* % of root-canal-filled teeth within this age category.

### Distribution of root canal fillings by tooth groups

Root canal fillings were registered in all tooth groups, with the largest tooth group being molars (41.4% of all root-canal-filled teeth) (see Table 3). Of the root-canal-filled teeth, 58.0% (*n* = 171) were positioned in the maxilla, and 42.0% (*n* = 124) in the mandible.

**Table 3:** Distribution of root canal fillings in different tooth groups according to jaw, presence of apical periodontitis (AP) and root canal filling quality.

Variable	Molars *n* (%)	Premolars *n* (%)	Incisors and canines *n* (%)
Total	122 (100.0)	109 (100.0)	64 (100.0)
Jaw			
Maxilla	57 (46.7)	59 (54.1)	55 (85.9)
Mandible	65 (53.3)	50 (45.9)	9 (14.1)
AP			
Yes	57 (46.7)	25 (22.9)	12 (18.8)
No	65 (53.3)	84 (77.1)	52 (81.3)
Root canal filling quality			
Adequate	21 (17.2)	42 (38.5)	26 (40.6)
Inadequate	101 (82.8)	67 (61.5)	38 (59.4)

### Observer variation

The intra-observer agreement on periapical status was substantial for both observers: 0.721 and 0.705, respectively. When comparing the assessment of periapical status on the first 121 root-canal-filled teeth, there was an initial difference in the assessment of 12 teeth that were discussed about until agreement. The inter-observer agreement on root canal filling quality was 0.686 (substantial) according to Cohen’s Kappa after individual assessments of the 30 root-canal-treated teeth but before the observers discussed their assessments to reach consensus.

### Prevalence of AP in root-canal-filled teeth

AP was present in 94 (31.9%) root-canal-filled teeth. For the prevalence of AP within tooth groups, see Table 3.

### Root canal filling quality

Of the 295 root-canal-filled teeth, 89 (30.2%) had an adequate root canal filling. For the frequency of adequate root canal fillings within tooth groups, see Table 3.

### Root canal filling quality in relation to AP

Of the total 206 teeth with technically inadequate root canal filling quality, 72 (35.0%) had AP, and of the 89 teeth with technically adequate root canal filling quality, 22 (24.7%) had AP.

### Results restricted to 20–70-year-olds

Since the earlier cross-sectional studies conducted in Jönköping (1973–2013) did not include root canal fillings among 80-year-olds, separate calculations were made for a subsample without 80-year-olds (see Table 4 for comparison with previous cross-sectional studies). The new distribution of root canal fillings was as follows: 43.4% molars (*n* = 85), 34.7% premolars (*n* = 68) and 21.9% incisors and canines (*n* = 43). The prevalence of AP in root-canal-filled teeth was 34.7%, with 80-year-olds being excluded and the frequency of technically adequate root canal fillings was 30.1%. For detailed information about tooth groups, see Table 5.

**Table 4:** Comparison between different years of the Jönköping study.

	Year					
	1973	1983	1993	2003	2013	2023***
Total number of root-canal-filled teeth analysed, *n*	1217	1169	984	611	491	196
AP in root-canal-filled teeth, %	24.5	23.8	21.1	24.6	34.0	34.7
Number of root-canal-filled molars, *n* (%)	210 (17.3*)	261 (22.3)	277 (28.2)	204 (33.4)	202 (41.1)	85 (43.4)
AP in root-canal-filled molars, *n* (%)	64 (30.5**)	62 (23.8)	67 (24.2)	65 (31.9)	93 (46.0)	43 (50.6)

AP: apical periodontitis.

* % of the total number of root-canal-filled teeth that year.

** % of the number of root-canal-filled molars that year.

*** The information in column 2023 is with only 20–70-year-olds included.

**Table 5:** Distribution of root canal fillings in different tooth groups according to jaw, presence of apical periodontitis (AP) and root canal filling quality when only 20–70-year-olds were included.

Variable	Molars *n* (%)	Premolars *n* (%)	Incisors and canines *n* (%)
Total	85 (100.0)	68 (100.0)	43 (100.0)
Jaw			
Maxilla	48 (56.5)	39 (57.4)	38 (88.4)
Mandible	37 (43.5)	29 (42.6)	5 (11.6)
AP			
Yes	43 (50.6)	17 (25.0)	8 (18.6)
No	42 (49.4)	51 (75.0)	35 (81.4)
Root canal filling quality			
Adequate	17 (20.0)	25 (36.8)	17 (39.5)
Inadequate	68 (80.0)	43 (63.2)	26 (60.5)

### Analysis of independent variables

The results of the GEE analysis can be seen in Table 6. Age, sex, tooth group, and the number of teeth were seen to have a statistically significant association with AP, with male sex, the tooth group molars, and a higher number of missing teeth having a higher risk for AP. The age groups 40 and 50 were also seen to have a higher risk for AP. Due to the high non-attendance rate in the youngest age groups, the association between age and AP could not be analyzed.

**Table 6:** A multiple General Estimation Equation (GEE) analysis for different variables with apical periodontitis (AP) being the dependent variable, with the level of significance set at 0.05.

Variable	Multiple GEE		
	Sig.	OR	95% CI
Sex			
Female	0.014	0.480	0.268–0.860
Male	reference	1	reference
Age			
20*		< 0.001	0.000–0.000
30*	0.091	13.675	0.658–284.197
40	0.005	4.367	1.553–12.280
50	0.007	3.154	1.361–7.313
60	0.966	1.029	0.278–3.804
70	0.089	1.903	0.906–3.993
80	reference	1	reference
Tooth group			
Molar	< 0.001	5.488	2.319–12.990
Premolar	0.194	1.636	0.779–3.434
Incisor/canine	reference	1	reference
Root canal filling quality			
Adequate	0.426	0.785	0.432–1.426
Inadequate	reference	1	reference
Number of root-canal-filled teeth	0.373	1.058	0.935–1.197
Number of teeth in the oral cavity	< 0.001	0.874	0.814–0.939

OR: odds ratio; CI: confidence interval.

In the analysis, the possible clustering effect has been taken into account by clustering the teeth based on individual but keeping the tooth as the unit of analysis.

* In age groups 20 and 30, the analysis could not be carried out correctly due to the low number of observations.

## Discussion

The data from the present cross-sectional study show a high frequency of AP in root-canal-filled teeth, as well as a large proportion of root canal fillings with inadequate root canal filling quality. This finding is especially evident in root-canal-filled molars that formed the largest tooth group in the sample.

In this study, 41.4% of all root-canal-filled teeth were molars when all age groups 20–80 were included and increased to 43.4% when 80-year-olds were excluded. The large proportion of root-canal-filled molars was also seen in the 2013 Jönköping study [11], and a sequential increase was also evident from 1973 to 2003 [12]. One explanation for this change could be the generally improved oral health in Sweden that has led to people retaining more teeth in higher ages [16]. Posterior teeth have been shown to present higher frequency of dental caries [23], which may eventually result in pulpal conditions requiring RCT. Endodontic treatment in molars is considered to be more technically complicated due to a more complex root canal anatomy, and a more posterior position in the oral cavity. While less than a fifth of the root-canal-filled molars had a technically adequate root canal filling quality, 40.6% of the incisors and canines, and 38.5% of the premolars had adequate root canal filling quality. Approximately half of the root-canal-filled molars in this study presented AP in intraoral radiographs, and the same phenomenon has been seen for example in a study by Laukkanen et al. [6], where 44.4% of the root-canal-filled molars had AP and 57.0% of root-canal-filled molars had inadequate root canal filling quality. It is therefore not surprising that in the multiple GEE analysis, the tooth group molars was considered to be associated with AP. At the same time, in the GEE analysis, the large proportion of molars may have affected the impact of other factors.

Many studies have emphasized the finding that a technically adequate root canal filling quality correlates positively with periapical healing [2, 3, 5]. One possible interpretation may be that root canal filling quality works as a surrogate variable for the quality of the endodontic treatment as a whole; for example, the length of the root canal filling is considered to correspond to the instrumentation length prior to obturation. However, neither the present study nor Silnovic et al. [11] could show a statistically significant association between root canal filling quality and AP. Even though there is a widespread support for the importance of root canal filling quality for periapical healing [2, 3, 5], the relation has been questioned in other studies. For example, Koch et al. [7] found that after introducing mechanical instrumentation to a group of dentists, the technical quality of root canal fillings, assessed in intraoral radiographs, showed a statistically significant improvement. However, in the same study, no statistically significant effect on the periapical healing was seen. It could therefore be questioned how well the presentation of a root canal filling in radiographs corresponds to all technical factors during the instrumentation, for example measures taken to ensure adequate asepsis and antiseptic treatment. In the present study, the high non-attendance rate may have contributed to insufficient statistical power, increasing the risk that a potential association between AP and poor root canal filling quality remained undetected.

An important difference in this study compared to the previous cross-sectional studies on AP and root canal filling quality in the Jönköping study series (1973–2013) was that the 80-year-olds were included [11, 12]. Previously, this age category was excluded from the analysis of root-canal-filled teeth. Older adults are a population subgroup that is becoming increasingly important in dentistry. The population in Sweden is aging, and people retain a higher proportion of their natural teeth with age [16]. Ageing increases the risk for poor oral hygiene and thus the risk for dental caries because of, for example, decreased salivary production, and change in the fine motor skills [17]. It is important to include this age group in order to monitor the development of oral health in the elderly. When the results of the present study were divided into all root-canal-filled teeth, and all root-canal-filled teeth except those from 80-year-olds, it could be seen that the results did not change substantially when it came to the proportion of tooth groups, frequency of AP or frequency of technically adequate root canal fillings (compare Tables 3 and 5). Even though risks of oral pathology increase with age, AP in root-canal-filled teeth is prominent in all age groups. In the multiple GEE analysis, it was seen that 40- and 50-year-olds had a statistically significantly higher risk for AP than root-canal-filled teeth in other age groups. This could be explained by the fact that older individuals had more missing teeth, and thereby teeth with AP may have been extracted and missing from the data. However, it cannot be excluded that the result may be due to chance since the number of root-canal-filled teeth among 40- and 50-year-olds was low. It should also be noted that root canal fillings may be underreported among 20-year-olds, as no intraoral radiographs of the anterior teeth were taken unless clinical pathology was present or a root canal filling was suspected.

In the GEE analysis, sex was seen to have a statistically significant association with AP, with women having lower risk for AP than men in the current sample. Similar results have been noted by Silnovic et al. [11], Frisk et al. [12], and Huumonen et al. [24]. The difference between men and women has been attributed to the differences in pain perception from root-canal-filled teeth with AP [25], but also the differences in health behavior, with the idea that women are more prone to seek dental care than men [26]. However, there is no consensus that sex per se would influence the periapical healing, and systematic reviews have discussed conflicting results [2, 5]. Another interesting finding is the association between number of teeth and AP in root-canal-filled teeth, with a higher number of retained teeth leading to a lower risk of AP. A higher number of retained teeth is usually associated with favorable oral health as well as young age.

Like cross-sectional studies in general, this study had limited information on the participants in comparison to clinical follow-up studies. There is no information on where, when and why the root canal fillings were conducted. The participants may have regularly attended public or private dental care in Sweden, but some of the treatments may have been done abroad. Therefore, there may be differences in the treatment methods that are not visible when looking at the intraoral radiographs. While the study sample is supposed to represent a randomized sample, due to the high non-attendance rate, it should also be asked which individuals decided to participate in the study: for example, in a study conducted in Taiwan [27], it was shown that people with more trust in biomedical research, and education in the area were more willing to participate in research projects. Similarly, people with certain attitudes towards dentistry and dental care may be more interested in participating in studies about oral health. However, this could not be assessed with the current sample because of the limited information about the non-attendants. Therefore, generalizations about the frequency of AP in root-canal-filled teeth and root canal filling quality in an adult normal population in Sweden should be made with caution. At the same time, the decreasing participation rate is a known phenomenon in epidemiological studies, and has been noticed globally [28, 29].

The limited information on the time and indication for the RCTs also limits the interpretations of the ‘true’ apical status of the root-canal-filled teeth. Some of the periapical lesions in the radiographs could be in the process of healing after a newly made root canal filling, while some of the lesions could be increasing in size due to remaining infection in the root canal system. However, Petersson et al. noticed that the number of periapical lesions healing or increasing in size was approximately the same [30]. Based on this, the eventual healing or worsening of the periapical condition does not need to be taken into account. However, since it cannot be said how many of the periapical lesions observed were under healing or worsening, it cannot be said how many of these may need treatment later.

Not only does the assessment of potential ongoing periapical processes has its limitations, but the diagnosis of AP in root-canal-filled teeth also presents challenges, particularly when it comes to determining where to draw the line between health and disease. The PAI, introduced by Ørstavik et al. [22], provides a standardized ordinal classification, ensuring reproducible classification and enabling inter-study comparisons. However, PAI constitutes a simplification of a continuous biological phenomenon into discrete categories with limited measurement resolution, introducing inherent potential for misclassification. In prevalence analyses, the PAI scores are highly sensitive to operational definitions, particularly the chosen threshold for dichotomization, which is often set between scores 2 and 3, as in the present study. Importantly, altering the dichotomization point can produce substantial changes in estimated prevalence and can materially affect comparative inferences between population groups and risk categories. The individual PAI scores can be of interest [see Table 1 Appendix], to see the distribution of data over the full index. In Table 2 Appendix, the frequency of AP in the present sample is altered using different cut-off points to show to which extent the study conclusions depend on the chosen cut-off point.

If all the root-canal-filled teeth in Sweden had the same frequency of AP as in the present study, that is, approximately one third, it would mean that 2.5 million root-canal-filled teeth would need to be treated [31]. However, this estimate is based on assumptions derived from the data in the present study, where the non-attendance rate was high. If these assumptions hold true, the resources of Swedish dentistry would be insufficient to treat all these teeth and current guidelines [1] may therefore not be practical. A large proportion of the patients with persistent AP in their root-canal-filled teeth are probably not aware of the disease [25, 32], that is, they have no sense of illness. However, at the same time, there is a group of individuals that have symptoms from their root-canal-filled teeth and have both subjective and objective need for further treatment, as well as individuals with systemic health conditions requiring retreatment or extraction to eliminate infections before starting certain medical treatments, for example chemotherapy. In Sweden, a large proportion of people attend regular dental checkups [33], and there is a large focus on preventive dental care. In the case of systemic diseases that can increase risks of complications such as sepsis, health care and dental care work together to minimize the risk of oral infectious foci negatively affecting general health [34, 35]. In the present study, the data from the clinical examinations or patient surveys were not used, and therefore, the proportion of participants in need for further treatment due to the periapical status of their root-canal-filled teeth could not be determined. Treatment methods with a predictable and more favorable outcome following RCT are preferable; but until then, a new strategy to predict which patients with AP in their root-canal-filled teeth are at high risk of worsening disease would enable more practical guidelines and efficient allocation of resources, benefiting both the individual and society. Development of such guidelines would help dentistry enabling a more uniform way of decision-making, based on the anamnestic, clinical and radiographic findings in each individual case; thus, such guidelines could help to better predict which patients would benefit the most from endodontic retreatment, endodontic microsurgery, extraction, or just monitoring of the periapical condition.

## Conclusion

This study sample demonstrated a high proportion of root-canal-filled teeth with AP and inadequate root canal filling quality, findings that were particularly pronounced in molars, which constituted the largest tooth group in the sample. These observations highlight the need for further investigation of their underlying causes, consequences, and potential avenues for improvement.

## Supplementary Material


