# Supplementary material for: Prevalence of apical periodontitis in root-canal-filled teeth – a cross-sectional study
Source: Acta Odontol Scand. 2026 Aug 13;85:46663. doi: 10.2340/aos.v85.46663 (PMC13483715; doi:10.2340/aos.v85.46663)
Supplement: Supplementary file 1 [file AOS-85-46663-s1.pdf]

Supplementary material has been published as submitted. It has not been copyedited or typeset by Acta Odontologica Scandinavica.

Table 1 Appendix. Distribution of PAI (periapical index) scores 1–5 and root canal filling quality between tooth groups.

	PAI					
	1	2	3	4	5	Total
Maxilla	100	26	35	6	4	171
Incisors and canines	36	10	6	3	0	55
Adequate	15	3	3	1	0	22
Inadequate	21	7	3	2	0	33
Premolars	40	9	10	0	0	59
Adequate	15	2	3	0	0	20
Inadequate	25	7	7	0	0	39
Molars	24	7	19	3	4	57
Adequate	6	3	1	0	0	10
Inadequate	18	4	18	3	4	47
Mandible	57	18	33	9	7	124
Incisors and canines	6	0	2	0	1	9
Adequate	2	0	1	0	1	4
Inadequate	4	0	1	0	0	5
Premolars	31	4	12	2	1	50
Adequate	13	3	5	0	1	22
Inadequate	18	1	7	2	0	28
Molars	20	14	19	7	5	65
Adequate	2	3	2	2	2	11
Inadequate	18	11	17	5	3	54

Table 2 Appendix. The frequency of AP (apical periodontitis) in the current sample changes, when the threshold for AP on the PAI (periapical index) scale is changed.

Threshold at PAI scale	PAI 1–2	PAI 2–3	PAI 3–4	PAI 4–5
Frequency of AP (%)	46.8	31.9	8.8	3.7
